# Pancreaticojejuno Anastomosis after Pancreaticoduodenectomy: Brief Pathophysiological Considerations for a Rational Surgical Choice

**DOI:** 10.1155/2012/636824

**Published:** 2012-03-05

**Authors:** Roberto Caronna, Nadia Peparini, Gabriele Cosimo Russillo, Adolfo Antonio Rogano, Giuseppe Dinatale, Piero Chirletti

**Affiliations:** General Surgery N, Sapienza University of Rome, Viale del Policlinico 155, 00161 Rome, Italy

## Abstract

*Introduction*. The best pancreatic anastomosis technique after pancreaticoduodenectomy (PD) is still debated. Pancreatic fistula (PF) is the most important complication but is also related to postoperative bleedings and pancreatic remnant involution. We support pancreaticojejuno anastomosis (PJ) advantages describing our technique with brief technical considerations. *Materials and Methods*. 89 consecutive patients underwent PD with suprapyloric gastric resection and double loop reconstruction. Pancreaticojejunal end-to-end anastomosis was done by simple invagination with a single layer of interrupted pledget-supported Ticron stitches. *Results*. Pancreatic fistula occurred in seven patients (7.8%): six cases of grade A fistula resolved spontaneously, and in only one case of grade B fistula percutaneous drainage was necessary. Postoperative hemorrhage occurred in only two (2.2%) of 89 patients. *Conclusion*. Pancreaticojejunostomy with minor changes in anastomotic techniques can contribute to improvement of the outcome of Roux-en-Y reconstruction regarding PF and other related complications. The particular reconstruction reported seems also to preserve the pancreatic exocrine function.

## 1. Introduction

The best pancreatic anastomosis technique after pancreaticoduodenectomy is still debated [1, 2]. Pancreaticojejunostomy is the commonly preferred method of anastomosis but the incidence of pancreatic fistula does not seem different according to the many techniques proposed for the reconstruction of pancreatic digestive continuity [3]. Other complications are also related to the onset of pancreatic fistula. The postoperative bleeding due to the erosion of peripancreatic vessels by the extravasated pancreatic juice has been described in 2–8% of cases [4–6] but morbidity rate increases from 6% to 26% when pancreatic fistula become manifest [7–9]. Finally, some authors believe that the involution of the residual pancreatic remnant is also related to the onset of the pancreatic anastomosis leakage [10].The authors report their experience with a particular PJ technique, evaluating the results on postoperative complications in a personal reconstruction modality after pancreaticoduodenectomy and making some pathophysiological considerations to support the advantages of PJ.

## 2. Materials and Methods

**Figure 1:**
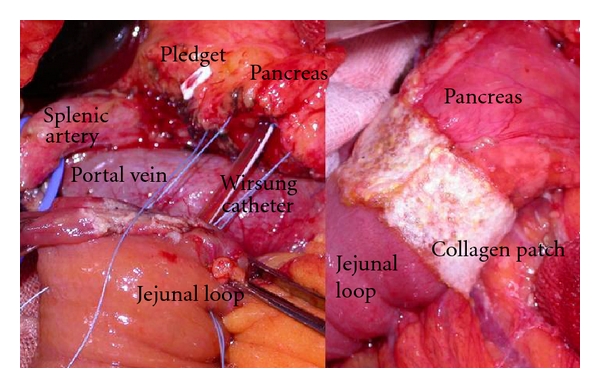
Roux-en-Y pancreaticojejuno anastomosis: pledgetsupported Ticron stitches between the seromuscularis of the jejunum and the pancreatic capsule, before the pancreatic invagination. A small catheter is inserted in the main pancreatic duct. On the right side, anterior aspect of pancreaticojejunal anastomosis after application of TachoSil.

We considered 89 consecutive patients who underwent PD at “La Sapienza” University (Rome, Italy) from January 1995 to June 2011. The mean age of patients was 60.8 years (range 35–85) (65.2% were males and 34.8% females). The underlying diseases were pancreatic carcinoma in 54 cases; pancreatic serous cystadenoma in six cases; mucinous cystadenoma in one case; pancreatic endocrine tumor in two cases; ampullar carcinoma in ten cases; distal bile duct carcinoma in twelve cases; chronic pancreatitis in three cases; a non-Hodgkin lymphoma of the pancreatic head in 1 case. In all patients, the surgical procedure comprised PD with suprapyloric gastric resection and Roux-en-Y reconstruction with anastomosis of the isolated Roux limb to the stomach and single Roux limb to both the pancreatic stump and hepatic duct. A meticulous hemostasis performed using bipolar coagulation and prolene 5/0 was carried out on the pancreatic section surface. In all patients the consistency of the pancreatic gland was noted as being soft or hard. Small catheter was inserted in the main duct, passed through the anastomosed bowel loop, and fixed to the abdominal wall (Figure 1). Wirsung duct size was evaluated according to the caliber of the catheter used for cannulation, considering it as normal when using a catheter No. 6 French and dilated when it was No. 8 French. A drainage tube was placed near the pancreaticojejunostomy; external biliary drainage was not used. Pancreaticojejuno end-to-end anastomosis was done by simple invagination of the pancreatic stump into the jejunal loop for 2 cm and suturing all around with a single layer of interrupted pledget-supported Ticron stitches between the seromuscularis of the jejunum and the pancreatic capsule (Figure 1). This establishes a close contact between the jejunal loop serosa and the pancreatic capsule. From January 2005, fibrinogen-/thrombin-coated collagen patch (TachoSil, Nycomed, UK Ltd.) has been also layered on suture line of pancreaticojejuno anastomosis (Figure 1) in the last 35 patients. All patients in our study received octreotide during the first six postoperative days. The postoperative surgical outcome within 60 postoperative days was assessed. PF, postoperative hemorrhage, and delayed gastric emptying (DGE) were assessed according to the International Study Group of Pancreatic Fistula and International Study Group of Pancreatic Surgery definitions [11]. 6 months postoperatively a group of 10 patients was subjected to assessment of pancreatic exocrine function reserve by evaluation of fecal elastase-1 concentration and by dynamic MR study of the pancreatic stump, with simultaneous stimulation with I.V. secretin injection (1 U/kg).

## 3. Results

The consistency of the pancreas was considered soft in 54 cases and hard in 35.A No. 6 Wirsung catheter was used in 29 cases and a No. 8 catheter in 60, corresponding to a normal or dilated Wirsung duct caliber.

PF occurred in seven patients (7.8%): six cases of grade A fistula resolved spontaneously, and in only one case of grade B fistula percutaneous drainage was necessary. In the 6 patients with a grade A pancreatic fistula, the pancreas was soft in 4 cases and hard in 2. In the single patient with grade B pancreatic fistula, the pancreatic consistency was soft.Regarding the size of the Wirsung catheter, the caliber was No. 6 in all patients who developed a pancreatic fistula. Mean age of patients with pancreatic fistula was 60.5 years.

Postoperative hemorrhage occurred in only two (2.2%) of 89 patients, biliary fistula in eight cases (8.9%), acute pancreatitis in one case (1.1%), and one patient with preexisting stenosis of hepatic artery developed thrombosis of the hepatic artery.

Grade A DGE occurred in eight patients (8.9%), grade C DGE in one patient (1.1%), left pleural effusion in 15 cases (16.8%), and wound infection in eight cases (8.9%).

Postoperative mortality rate was 2.2% (two out of 89 patients: acute myocardial infarction; sepsis due to acute pancreatitis).

Regarding the assessment of exocrine functional reserve of the pancreatic stump, in the 10 patients evaluated for pancreatic fecal elastase-1 and dynamic MR pancreatograms with secretin stimulation, only 2 cases showed fecal elastase-1 below 200 (normal range: 200–500 micrograms/g of feces) with a lack of dilation of the residual Wirsung duct and poor filling of the anastomosed pancreatic jejunal loop, documented by dynamic MR after secretin stimulation. The remaining 8 patients, on the other hand, showed an increase of Wirsung duct caliber with normal passage of pancreatic juice in the anastomosed jejunal loop after secretin stimulation and fecal elastase-1 concentration in the normal range.

## 4. Discussion

A recent meta-analysis [12] shows no statistically significant differences among different pancreatic reconstructions after PD.

Although a meta-analysis possibly provides the best methodology, it is usually limited by clinical heterogeneity. The lack of a uniform definition of PF and postoperative complications hampered the data analysis and resulted in clinical heterogeneity. The modified technique of pancreatic reconstruction after PD may also result in clinical heterogeneity. For example, PJ is performed as either an end-to-end anastomosis with invagination of the pancreatic stump into the jejunum or as an end-to-side anastomosis. A main pancreatic duct stent might be placed across these anastomoses. Thus, the overall heterogeneity of the available studies severely hampers conclusive comparisons, and therefore one must make interpretations with caution.

We believe with others that the successful management of pancreatic anastomoses may depend more on meticulous surgical techniques, surgical volume, and other management parameters rather than on the type of technique used [12]. But we also think that few considerations are very important about the choice of reconstructive procedure on postoperative complications and also on functional outcome of the pancreatic remnant after PD.

The invagination of pancreatic stump into the jejunal loop and the sealing effects of TachoSil layered on a pancreaticojejunal anastomosis may reduce the risk of the overflow of pancreatic juice from the anastomosis site during the first postoperative days and may minimize the risk of hemorrhage due to vessel erosion or development and bleeding of visceral arterial pseudoaneurysms caused by digestion of the arterial vessel wall near a pancreaticojejunal leak by trypsin and elastase [4–6]. The pancreatic juice that escapes from the lower ducts on pancreatic section line must be considered, and therefore we believe that the invagination of pancreatic stump can allow a physiological collection of the pancreatic juice into the jejunal loop, limiting its extravasation.

Carrying out PJ by invagination of the pancreatic stump into the jejunum and Ticron-pledgeted sutures makes a homogeneous anastomotic surface that supports the adhesion of TachoSil and optimizes the sealing effect. Pledgeted sutures make easy the pancreatic invagination even in cases of soft pancreatic parenchyma.

Similarly, we believe that it is useful to place a drain into the Wirsung duct in the first days after surgery because it brings out a large amount of the pancreatic juice. In case of anastomotic leakage the pancreatic juice that comes in contact with the dehiscent area is minimized. This hypothesis has been recently supported by others [13].

Regarding the pancreaticogastrostomy, more intraluminal postoperative hemorrhages and more severe exocrine insufficiency than PJ have been reported in different studies with significantly more severe atrophic changes in remnant pancreas [14]. Obstruction or stenosis of the pancreaticodigestive anastomosis has been suggested to be the cause of ductal dilatation and parenchymal atrophy after pancreaticoduodenectomy [15–17], and, as Tajima underlines, anastomotic stenosis may occur after pancreatic anastomosis leakage [10].

From this point of view and according to our experience, if the PJ shows a low incidence of PF often of grade A with a low incidence of hemorrhagic complications related to PF, we believe that PJ should be considered as an advantageous surgical choice.

The impact of reconstruction procedure other than the type of pancreaticoenteric anastomosis should also be considered in the evaluation of morphologic and functional changes of the remnant pancreas after pancreaticoduodenectomy.

Apart from anastomotic stenosis, involutional atrophy of the pancreatic remnant may also be a consequence of deregulation of pancreatic neurohormonal stimulatory factors resulting from pancreaticoduodenectomy [16, 18, 19].

Mori and colleagues demonstrated that pancreatic dysfunction was due not only to gastrectomy and duodenectomy resulting from pancreaticoduodenectomy, but was also related to treatment of the pancreatic remnant [20].

The pancreatic remnant may then be able to preserve its exocrine and endocrine function if its physiologic stimuli are maintained.

According to our previous works [21], we believe that two conditions are very important in the maintenance of pancreatic exocrine function after pancreaticoduodenectomy. First, gastric preservation favours adequate weight gain after surgery due to higher caloric intake. Normal acid secretion acts as a physiologic stimulus on the duodenal mucosa, promoting the secretion of secretin and CCK-PZ [22], as well as the subsequent stimulation of pancreatic exocrine secretion with better digestion of protein and fat (weight gain). Second, after removal of the duodenal source of CCK and secretin, preservation of the first jejunal loop in the reconstruction of the alimentary circuit maintains the physiologic jejunal secretion of secretin and CCK-PZ subsequent to alimentary transit and can compensate (at least in part) for the abolished duodenal hormonal release.

Because of the elimination of the entire duodenum, basal concentrations of plasma CCK are significantly lower in patients who undergo pancreatoduodenectomy than in preoperative patients, and postprandial plasma secretin concentrations are significantly lower in patients who undergo the Whipple procedure than in control patients or patients treated by pylorus-preserving pancreaticoduodenectomy in which the entire stomach and duodenal bulb are preserved. Moreover, reduced (but significant) amounts of CCK and secretin are released postprandially even after pancreaticoduodenectomy, suggesting a compensatory mechanism of the remnant of the upper small intestine: in fact, after the duodenum, the upper jejunum is the second most important source of release of CCK and secretin [23, 24].

In our technique of pancreaticoduodenectomy, we adopted a suprapyloric gastric resection (i.e., subtotal stomach preserving) and Roux-en-Y reconstruction with anastomosis of the isolated Roux limb (i.e., first jejunal loop) to the stomach and single Roux limb (i.e., second jejunal loop) to the pancreatic stump and hepatic duct [25]. According to these assumptions, we observed, by dynamic MR pancreatograms obtained by secretin injection, that the patency of the pancreaticojejunal anastomosis was evident in all patients evaluated [21]. In the same group, the concentration of fecal-1 elastase decreased only in the two patients with reduced jejunal filling detected by dynamic MR [21].

## 5. Conclusion

Pancreaticojejunostomy with minor changes in anastomotic techniques can contribute to improvement of the outcome of Roux-en-Y reconstruction regarding PF and other related complications. The particular reconstruction reported seems also to preserve the pancreatic exocrine function as detected by MR pancreatography with secretin stimulation and fecal 1-elastase assay.
